# Impact of Cataract on Color Vision and Contrast Sensitivity: A Clinical Review

**DOI:** 10.7759/cureus.105241

**Published:** 2026-03-14

**Authors:** Mansour A Alghamdi

**Affiliations:** 1 Optometry Department, College of Applied Medical Sciences, King Saud University, Riyadh, SAU

**Keywords:** cataract, color vision, contrast sensitivity, lens opacification, visual function

## Abstract

This clinical review examines current evidence on the effects of cataracts on color vision and contrast sensitivity (CS), the underlying optical mechanisms, and functional outcomes following cataract surgery. Evidence was synthesized from structured searches of the PubMed and Scopus databases, covering database inception to January 2026. Included studies quantitatively assessed how age-related cataract type or lens opacity density affects color vision, CS, and functional visual outcomes before and after standard phacoemulsification with posterior chamber intraocular lens implantation. Cataracts consistently impair color vision, most notably along the blue-yellow (tritan) axis, particularly in nuclear cataracts, likely because of selective short-wavelength light absorption by the yellowed crystalline lens. With increasing lens opacity density, additional red-green (protan) deficits may also occur. CS is reduced, particularly at intermediate spatial frequencies around 3-6 cycles per degree, likely in association with increased intraocular light scatter. These functional deficits may appear in early stages and worsen with disease progression. Cataract surgery generally results in rapid and clinically meaningful improvements in color discrimination, CS, glare tolerance, and vision-related quality of life. Overall, the reviewed evidence indicates that cataracts have a substantial impact on visual function beyond visual acuity and that assessment of color vision and CS provides important additional insight into cataract-related visual disability.

## Introduction and background

Cataract, the opacification of the crystalline lens, remains the leading cause of blindness and a major contributor to visual disability worldwide, representing a substantial and growing public health burden, particularly in aging populations and low-resource settings [1-7]. Although high-contrast visual acuity is the most commonly assessed metric for visual impairment in patients with cataracts, this single measure fails to capture the full spectrum of functional visual dysfunction experienced in daily life [8,9].

Critical components of visual performance, including color vision and contrast sensitivity (CS), are profoundly affected by cataracts and often correlate more closely with patients’ subjective complaints and real-world functional difficulties than Snellen acuity alone [8-12].

Beyond acuity loss, impairment of color discrimination and CS has important consequences for everyday functioning and safety. Patients frequently report difficulties with reading, driving at night, recognizing faces, navigating dim or low-contrast environments, and detecting hazards, which may compromise independence and increase the risk of accidents, particularly among older adults [12-14]. From a clinical perspective, these functional limitations influence patient-reported outcomes, timing of surgical referral, and postoperative satisfaction, emphasizing the need for broader functional assessment strategies in cataract management [12,15].

This review synthesizes current clinical and experimental evidence regarding the effects of cataracts on color vision and contrast sensitivity, the underlying optical mechanisms responsible for these deficits, and the extent of functional recovery following cataract extraction with intraocular lens implantation. A clearer understanding of these multidimensional visual changes is essential for optimizing clinical evaluation, guiding surgical decision-making, and improving patient-centered outcomes and quality of life after surgery [16-18].

This clinical review integrates evidence on both color vision and contrast sensitivity and discusses their combined implications for cataract-related visual disability, postoperative functional recovery, surgical timing, and intraocular lens selection.

## Review

Methods

This comprehensive review synthesizes the clinical and experimental evidence on the effects of cataracts on color vision and contrast sensitivity (CS). A structured literature search was conducted using PubMed and Scopus databases, supplemented by manual screening of reference lists to identify relevant peer-reviewed studies published in English. The search strategy covered database inception to January 2026, and the search terms included combinations of cataract, age-related cataract, lens opacity, color vision, contrast sensitivity, visual function, and intraocular lens.

Studies were considered eligible if they investigated (1) the impact of age-related cataract type or lens opacity density on color vision and/or CS, or (2) functional visual outcomes before and after cataract surgery with intraocular lens (IOL) implantation. Emphasis was placed on studies that employed standardized cataract grading systems, including the Lens Opacities Classification System III (LOCS III), and validated psychophysical assessments of color vision (CS). Non-English articles, single case reports, conference abstracts without full data, and studies in which non-cataract ocular pathologies were the primary cause of visual dysfunction were excluded.

Titles and abstracts retrieved from the database search were screened for relevance, followed by a full-text review of potentially eligible studies. When multiple publications reported overlapping patient populations or highly similar methodologies, the most comprehensive or recent study was prioritized. Considering the heterogeneity of cataract morphologies, grading methods, and visual function testing protocols across the literature, a narrative synthesis approach was adopted rather than a formal systematic review or meta-analysis. This approach enabled integrated interpretation of findings across diverse methodologies, focusing on clinically meaningful patterns, underlying optical mechanisms, and functional implications for patient care.

All visual assessment tools discussed in this review are cited for descriptive and academic purposes only. No proprietary instruments, scales, questionnaires, or scoring systems were administered directly by the author. The tools referenced are commercially available clinical assessments and are described according to their original peer-reviewed publications. No copyrighted materials were reproduced as part of this review.

Epidemiology and classification

Global Burden of Cataract

Cataracts remain a leading cause of preventable visual impairment worldwide, contributing substantially to both blindness and moderate-to-severe visual impairment (MSVI), particularly among older adults. Global estimates indicate that cataracts represent one of the most common causes of MSVI, with tens of millions affected worldwide. Cataract burden is expected to increase due to population aging and unequal access to timely surgical services [2-6]. In addition to their contribution to blindness, cataracts significantly impair distance vision and daily functioning [19].

Classification of Cataracts

The severity and type of cataracts were graded using the Lens Opacities Classification System III (LOCS III), a standardized and widely validated tool for evaluating nuclear opalescence, nuclear color, cortical, and posterior subcapsular (PSC) cataracts on a decimal severity scale [20,21]. Progressive worsening of lens opacity, as measured by LOCS III, correlates strongly with declines in visual acuity and other functional vision parameters. This classification is important because different cataract morphologies selectively affect specific aspects of visual function.

Cataract morphology plays a critical role in determining the nature and severity of visual dysfunction. Nuclear sclerotic cataracts are characterized by progressive hardening and yellowing of the lens nucleus and exert the most pronounced effects on color vision through selective attenuation of short-wavelength light. Cortical cataracts consist of spoke-like opacities within the lens cortex and primarily impair contrast sensitivity by increasing intraocular light scatter and glare. Posterior subcapsular cataracts are located near the posterior pole of the lens along the visual axis and are associated with disproportionate visual disability, including marked glare sensitivity, reduced contrast sensitivity, and impaired near vision. The LOCS III system, therefore, facilitates meaningful correlation between cataract type, severity, and functional visual outcomes.

The application of standardized grading systems not only guides clinical decision-making regarding surgical timing but also improves comparability across studies and supports more precise interpretation of functional visual changes associated with specific cataract morphologies.

Mechanisms of visual impairment

Effects of Cataract on Color Vision

Cataracts induce systematic, wavelength-dependent alterations in color discrimination, a critical aspect of visual function that is often overlooked in standard clinical assessments. The primary mechanism underlying this impairment is the progressive opacification and yellowing (brunescence) of the crystalline lens, which functions as a short-pass filter, selectively absorbing and scattering incoming light, particularly at the blue-violet end of the spectrum [22]. This selective attenuation disproportionately reduces the stimulation of short-wavelength-sensitive cones (S-cones), leading to an acquired color vision deficit primarily along the tritan (blue-yellow) axis [23,24].

Psychophysical testing, such as the Farnsworth-Munsell 100-Hue test and the high-definition Cone Contrast Test (CCT), consistently demonstrates elevated tritan-axis error scores that correlate with cataract severity, particularly LOCS III nuclear grade [22,23,25,26]. Nuclear sclerotic cataracts exert the strongest chromatic effects as the progressive accumulation of chromophores in the nucleus selectively reduces short-wavelength transmission [27]. Patients often report subjective symptoms, including muted blue hues, yellowish color bias, and reduced chromatic contrast. Although cortical cataracts typically produce milder chromatic distortions stemming from scatter-mediated degradation, PSC may induce glare-dependent color discrimination errors, especially in bright light [11].

Importantly, studies using CCT have quantified cone-specific deficits, demonstrating that S-cone sensitivity declines most rapidly with age and is particularly affected by cataracts [22,23]. Following cataract extraction, the CCT scores for S-cones exhibited the most significant increase, confirming that these color vision deficits were primarily optical rather than retinal or neural in origin [22,23].

In addition to quantitative losses in color discrimination, cataract-related chromatic deficits have important functional implications for daily visual tasks. Impaired blue-yellow discrimination affects color-dependent activities such as reading color-coded information, recognizing traffic signals under variable illumination, and distinguishing objects in low-contrast environments. These deficits may be particularly problematic for older adults, in whom age-related neural changes may coexist with optical degradation [12,14,26]. Importantly, several studies demonstrate that color vision impairment may be present even when visual acuity remains relatively preserved, underscoring the clinical value of chromatic assessment as a complementary measure in cataract evaluation.

In addition to the optical effects of lens brunescence, it is important to distinguish cataract-related, acquired color vision deficits from congenital color vision deficiencies. Congenital red-green anomalies typically present early in life, are nonprogressive, and follow characteristic protan or deutan confusion axes, whereas cataract-related deficits emerge later, progress with increasing lens opacity, and predominantly affect blue-yellow discrimination [10,22-24]. This pattern supports an acquired, wavelength-dependent filtering mechanism rather than a primary retinal or genetic cone dysfunction. A comprehensive review of acquired color vision deficiencies confirms that the tritan axis is preferentially affected in media opacities such as cataracts, whereas retinal and optic nerve disorders typically produce more complex patterns involving multiple axes [28].

Recent work has also explored the dynamics of color perception immediately after cataract surgery. Removal of a heavily yellowed crystalline lens and implantation of a clear IOL can lead to a transient shift in color appearance, with some patients describing a temporary exaggeration of blue hues or a brief period of "temporary tritanopia" as the visual system adapts to the sudden increase in short-wavelength light [29]. Longitudinal measurements, however, indicate that color discrimination typically normalizes within weeks to months after surgery, and most patients ultimately experience improved chromatic contrast and more natural color perception relative to their preoperative status [10,22,23,29].

These findings emphasize that both static and dynamic aspects of color vision should be considered when counseling patients about expected postoperative visual changes. Providing realistic preoperative education about potential transient color shifts may help align patient expectations and improve satisfaction with surgical outcomes [29]. Table 1 summarizes key clinical studies detailing the impact of cataracts on color vision.

**Table 1:** Summary of key studies on cataract and color vision impairment PSC: posterior subcapsular; LSR: linearly scored results

	N (Eyes / patients)	Cataract type / grading	Color vision test	Key indices / main findings
Mehta et al. [22]	355 eyes; 25 patients (pre/post-op)	Age-related cataract (phakic vs pseudophakic comparison)	High-Definition Cone Contrast Test (CCT) or ColorDx CCT HD)	Cataract significantly reduced cone contrast sensitivity, disproportionately affecting S-cone–mediated vision. Cataract extraction produced the greatest improvement in S-cone CCT scores (~33 units), confirming an optical origin of the tritan-like deficit.
Jolly et al. [10]	50 patients (39 completed post-op)	Age-related cataract (LOCS III; mixed nuclear, cortical, PSC)	Low-Vision Cambridge Colour Test (CCT)	Cataract significantly reduced color discrimination, predominantly along the tritan axis, with additional protan deficits. Cataract surgery resulted in significant postoperative improvement in color vision, with nuclear cataract demonstrating the greatest preoperative impairment.
Langina-Jansone et al. [24]	Sample size not specified	General Cataract	Saturated Farnsworth D-15 arrangement test (with Bowman, Vingrys & King-Smith, and LSR analyses)	Cataract reduced chromatic resolution and produced predominantly tritan-like error patterns preoperatively. Following cataract surgery, most patients achieved normal color vision, although mild residual tritan and protan defects persisted in some cases.
Friström & Lundh [23]	30 eyes (30 patients)	Cortical, nuclear sclerosis, and PSC cataracts; comparison with pseudophakia	Computerized color contrast sensitivity testing (protan, deutan, tritan axes)	Cataract significantly impaired color contrast sensitivity, most prominently along the tritan (blue-yellow) axis. Color contrast sensitivity improved following cataract surgery, although pseudophakic eyes did not fully reach normal levels.
Ao et al. [27]	60 eyes (30 cataract; 30 control)	Age-related cataract; clinical grading	Farnsworth–Munsell 100-Hue test (photopic and mesopic conditions)	Age-related cataracts significantly impaired color discrimination under both photopic and mesopic conditions. Cataract surgery effectively restored color discrimination to normal levels.

Effects of Cataract on CS

CS is a highly sensitive indicator of cataract-related visual impairment that often declines before a measurable loss in visual acuity [8,29]. The combined effects of forward light scattering reduced retinal illuminance, and optical blur disproportionately impaired CS, especially at intermediate and high spatial frequencies (SFs), typically in the range of 3-6 cycles per degree (cpd) [8,12]. This range is critical for real-world tasks, such as night driving, face recognition, and object detection under low-contrast conditions [16]. The impact on CS varies significantly according to cataract morphology. Nuclear cataracts cause a moderate generalized loss of CS, often concentrated at higher SFs [11]. In contrast, cortical cataracts produce more substantial reductions due to refractive irregularities and increased glare susceptibility [11]. PSC causes the most severe and earliest CS deterioration owing to its central location on the visual axis, often resulting in pronounced deficits at low spatial frequencies [11,19]. Quantitative assessments, including the Pelli-Robson chart, SPAReCS test, and novel Quantitative Contrast Sensitivity Function (qCSF), consistently demonstrate that CS decline correlates more closely with patient-reported visual disability than with Snellen visual acuity [9]. In particular, the qCSF method is highly effective in detecting disproportionate deficits at 6 cpd in patients with cataracts, a spatial frequency range known to be strongly associated with functional visual disability in cataract patients [30]. Additional studies confirm that the severity of lens opacity, as graded by LOCS III, correlates linearly with reductions in CS across multiple spatial frequencies [11,31]. Early psychophysical studies established that glare disability in cataract patients results from forward light scatter and can be quantified as a selective impairment of the CS function under bright background illumination [32]. The reduction in CS associated with cataracts has substantial consequences for real-world visual performance. Tasks such as night driving, face recognition, mobility in dim environments, and hazard detection rely heavily on intermediate spatial frequencies rather than high-contrast detail. As a result, patients with early cataracts may experience significant functional limitations despite maintaining relatively good Snellen visual acuity. CS testing, therefore, provides a more ecologically valid assessment of visual disability and has been shown to correlate more strongly with patient-reported outcomes and quality-of-life measures than visual acuity alone.

The contrast sensitivity function (CSF) provides a more comprehensive description of visual performance than single-point measures such as Pelli-Robson scores or high-contrast acuity. In cataract patients, qCSF and related psychophysical paradigms consistently reveal disproportionate losses at mid-spatial frequencies around 3-6 cycles per degree, which are most relevant to everyday tasks such as face recognition, reading under suboptimal illumination, and detecting low-contrast obstacles [3,8,9,12]. These selective deficits can remain undetected by conventional acuity charts, thereby underestimating the true burden of disease [8,12].

Guidelines and expert opinion increasingly support the integration of functional measures, including CS, glare testing, and patient-reported outcomes, into cataract assessment and surgical planning [12,14,15]. Such an approach is particularly valuable in patients whose subjective complaints are disproportionate to their measured visual acuity, in whom CS loss may be the primary driver of disability and a key indication for earlier surgical intervention [8,12-14]. Table 2 summarizes the key studies on the effects of cataracts on CS.

**Table 2:** Summary of key studies on cataract and contrast sensitivity impairment CS: contrast sensitivity; PSC: posterior subcapsular; VA: visual acuity; LOCS III: Lens Opacities Classification System III; cpd: cycles per degree

Study (year)	N (Eyes / patients)	Cataract type / grading	Test method	Key indices / main findings
Vingopoulos et al. [8]	167 eyes (58 cataract, 77 controls, 32 pseudophakic)	Cataract, general	Quantitative Contrast Sensitivity Function (qCSF)	qCSF detected significant contrast sensitivity deficits in cataract eyes, with disproportionate impairment at intermediate spatial frequencies (~6 cpd).
Shandiz et al. [11]	135 eyes	Nuclear, cortical, PSC (LOCS III)	CSV-1000E (with and without glare)	CS without glare was reduced in all types. PSC caused the most severe CS loss. CS correlated better with patient-reported visual disability than VA.
Elliott et al. [12]	Sample size not specified	Nuclear, cortical, PSC	Arden plates, Pelli–Robson	CS decline is primarily an intermediate and high spatial frequency loss. PSC caused the most severe CS loss, especially at low spatial frequencies. Glare sensitivity increased for all types.
Rubin et al. [14]	72 patients (pre- and post-op)	Early cataract	Acuity, CS, glare sensitivity	CS and glare sensitivity are more sensitive to the effects of intraocular light scatter than VA. CS loss was greater at higher spatial frequencies.
Chylack et al. [20]	Sample size not specified	Nuclear, cortical, PSC (LOCS III)	Contrast sensitivity function	CS function was decreased only by nuclear color. CSF testing provided no more information about cataract-related visual loss than VA in early stages.

Functional Impact on Daily Activities

Cataract-related losses in color vision and CS have substantial consequences for everyday visual tasks that extend beyond the measurement of high-contrast visual acuity. Patients frequently report difficulties with night driving, walking in dimly lit environments, detecting curbs or steps, and recognizing facial expressions at a distance, even when Snellen acuity remains relatively preserved [8,12-14]. These functional complaints are closely linked to deficits at intermediate spatial frequencies, particularly in the range of 3-6 cycles per degree, which support tasks such as object detection, hazard recognition, and reading road signs under low-contrast conditions [8,12,13].

Impairment of color vision, especially along the blue-yellow axis, can further compromise performance in color-coded environments. Patients may struggle with distinguishing traffic lights and signs under adverse weather conditions, interpreting color-coded medication labels, or identifying colored wiring and indicators in occupational settings [10,12,22]. In older adults, these optical deficits may compound age-related neural changes, thereby amplifying the impact on functional independence and increasing the risk of falls and mobility-related accidents [12,14].

These findings are consistent with reports that functional measures such as CS and color vision may better reflect patient-reported visual disability than visual acuity alone [2,3,8,11,16,19,33].

Clinical findings and surgical outcomes

Differential Effects of Cataract Types

The specific morphology of cataracts determines their primary impact on visual function. A summary of the differential effects is presented in Table 3.

**Table 3:** Differential effects of cataract types and severity on visual functions LOCS III: Lens Opacities Classification System III

Cataract type	LOCS III grading domain	Visual acuity effect	Contrast sensitivity effect	Color vision effect	Notes
Nuclear cataract [21,22,27]	Nuclear color and opalescence	Progressive decrease with increasing severity	Moderate decrease, especially mid spatial frequencies	Predominant decline along the blue–yellow (S-cone / tritan) axis	Most severe effect on color vision
Cortical cataract [11,12]	Cortical opacities	Decrease mainly with increasing opacity	Significant reduction with glare, high frequencies	Mild-moderate effect on color vision	Causes glare and light scatter
Posterior subcapsular cataract [11,19]	Posterior subcapsular opacities	Notable visual acuity and glare-related impairment	Severe contrast loss due to central visual axis involvement	Glare-related color vision impairment	Causes glare disability, affects reading vision

The differential effects of cataract morphology on visual function highlight the limitations of a uniform approach to cataract assessment and management. Nuclear cataracts predominantly compromise chromatic discrimination through wavelength-dependent absorption, whereas cortical and posterior subcapsular cataracts exert a greater influence on CS and glare-related disability. These distinctions support the need for individualized functional assessment strategies that take into account cataract type and patient-specific visual demands. Incorporating CS and color vision testing into routine evaluation may therefore aid in optimizing surgical timing and aligning clinical decisions with patient-reported symptoms.

Functional Recovery Following Surgery

Across the clinical studies summarized in this review, cataract extraction was generally performed using standard phacoemulsification with posterior chamber intraocular lens (PCIOL) implantation, unless otherwise specified in the original reports. Cataract extraction with IOL implantation is highly effective, substantially improving both chromatic discrimination and CS, restoring visual function that supports daily activities and enhances the quality of life [19,29,16,34]. Postoperative studies have consistently demonstrated the rapid recovery of color vision and CS, substantiating the optical basis of preoperative impairments. Photostress recovery tests also demonstrated improved retinal and neural function following surgery, indicating the restoration of both optical clarity and downstream processing [29]. Longitudinal studies with extended postoperative follow-up confirm that improvements in color discrimination are sustained long-term, with most patients achieving normal or near-normal chromatic thresholds within three months of surgery [34,35].

Clinical Relevance of Functional Testing

A multidimensional assessment of visual function may be helpful when determining the optimal timing of cataract surgery. Patients may present with relatively good Snellen acuity yet report disabling glare, difficulty driving at night, or problems functioning in low-contrast environments, all of which are better captured by CS and glare testing than by standard visual acuity charts [8,9,12,13]. In such cases, documenting reduced CS or marked glare sensitivity may provide additional objective support for surgery and facilitate more accurate certification of visual disability [8,12,14].

Color vision testing likewise has an important role in selected clinical scenarios. Marked tritan deficits that improve after cataract extraction support an optical etiology and may help distinguish cataract-related changes from retinal or optic nerve pathology [10,22,24,27,29]. Incorporating both CS and color vision assessment into routine cataract evaluation may help align surgical decisions with patient-reported symptoms and potentially improve postoperative satisfaction and quality-of-life outcomes [16-19]. In addition, preoperative functional findings, particularly when CS is markedly reduced, may also be relevant when selecting intraocular lens type, as patients with substantial contrast loss may be less suitable for lens designs associated with reduced postoperative contrast performance.

The Role of IOLs

The selection of IOL can also influence postoperative visual outcomes. A significant area of research has been the comparison of yellow-tinted (blue-light-filtering) IOLs with standard clear ultraviolet-blocking IOLs. The rationale for using yellow-tinted IOLs is to mimic the natural protection of aging crystalline lenses, potentially reducing chromatic aberration and phototoxicity. Although some studies report marginal improvements in CS and reduced glare under certain photopic and mesopic conditions with yellow-tinted IOLs, most large-scale investigations have not demonstrated clinically meaningful differences in visual acuity, color discrimination, or overall subjective visual quality when compared with clear IOLs [16,36-41]. Postoperative functional outcomes may also vary according to optical design characteristics of implanted lenses, as studies evaluating multifocal diffractive intraocular lenses have demonstrated associations between contrast sensitivity performance and patient-reported quality-of-life improvements following cataract surgery [42]. Table 4 provides a summary of the key studies in this area.

**Table 4:** Summary of key studies comparing yellow-tinted and clear intraocular lenses UV: ultraviolet; cpd: cycles per degree

Authors (year)	N (eyes / patients)	IOLs compared	Methods used	Key indices / main findings
Hayashi & Hayashi [37]	148 eyes (148 patients)	Yellow-tinted IOLs vs non-tinted IOLs	Visual acuity, contrast-related measures, and glare assessment	No clinically significant differenceswere observed in visual function, including visual acuity or glare performance, between yellow-tinted and non-tinted IOLs.
Rodríguez-Galietero et al. [38]	44 eyes (44 diabetic patients)	AcrySof Natural (SN60AT, yellow) vs AcrySof SA60AT (UV-only)	CSV 1000-E, FM-100 Hue	Slight improvement in contrast sensitivity and blue-yellow discrimination in diabetic eyes with yellow IOLs; findings are limited to the diabetic population.
Rodríguez-Galietero et al. [39]	40 eyes (40 diabetic patients)	AcrySof Natural (SN60AT, yellow) vs AcrySof SA60AT (UV-only)	CSV-1000E, FM-100 Hue	Higher contrast sensitivity at selected spatial frequencies (3–18 cpd) with yellow IOLs; no differences noted in color vision or patient satisfaction.
Leibovitch et al. [40]	19 eyes (9 yellow, 10 clear)	AcrySof Natural (SN60AT, yellow-tinted; 9 eyes) vs AcrySof single-piece (SA60AT, UV-only; 10 eyes)	Visual acuity assessment; contrast sensitivity testing; color perception evaluation under photopic and mesopic conditions	Postoperative visual outcomes, including visual acuity, contrast sensitivity, and color perception, were comparable between yellow-tinted and UV-only intraocular lenses, with no clinically significant functional advantage demonstrated for the yellow-tinted IOL.
Cionni & Tsai [41]	54 eyes (54 patients)	AcrySof Natural (yellow-tinted) vs AcrySof single-piece (UV-only)	FM 100-Hue under photopic and mesopic conditions	No detectable difference in color perception between AcrySof Natural and UV-only AcrySof single-piece intraocular lenses under either lighting condition.
Kara-Junior et al. [16]	60 eyes (60 patients; 5-year follow-up)	Blue-light–filtering (yellow-tinted) IOLs vs UV-only (clear) IOLs	Long-term (5-year) follow-up; assessment of color perception, photopic and scotopic contrast sensitivity, and macular status	After long-term follow-up, no significant differences were observed in color perception or photopic/scotopic contrast sensitivity between blue-light–filtering and UV-only IOLs; any potential macular protective effect remained inconclusive.

From a clinical perspective, the functional differences reported between yellow-tinted and clear IOLs appear small and inconsistent in routine cataract practice. Current evidence, therefore, does not support a generalized functional advantage of blue-light-filtering IOLs with respect to postoperative color vision or CS [16,36-41]. Instead, IOL selection is more likely to depend on individual patient characteristics, including visual demands, tolerance for dysphotopsias, coexisting retinal status, and the relative importance of maximizing postoperative contrast performance.

In addition to their spectral filtering properties, contemporary IOL designs increasingly aim to optimize the balance between extended depth of focus and preservation of CS. Enhanced monofocal and extended-depth-of-focus (EDOF) lenses can improve intermediate vision and reduce spectacle dependence, but some designs may be associated with small reductions in CS or increased dysphotopsias compared with standard monofocal IOLs [36-38,43,44]. Recent comparative studies of enhanced monofocal IOLs report favorable intermediate vision outcomes with minimal compromise in photopic CS under standardized testing conditions [43-46]. These trade-offs may be particularly relevant in patients who already exhibit substantial preoperative CS loss, in whom maximizing postoperative contrast performance may be prioritized over spectacle independence [43-46].

Emerging evidence also suggests that postoperative CS and patient-reported visual quality can vary between different aspheric and aberration-correcting IOL platforms, especially under mesopic and glare conditions [43-46]. In this context, preoperative CS and visual demands may provide useful additional information when selecting between standard monofocal, enhanced monofocal, and EDOF designs. By contrast, multifocal IOLs may be associated with greater reductions in CS and higher rates of dysphotopsias, particularly in patients with pre-existing visual dysfunction [47]. Future studies using standardized CS and color vision outcomes are needed to clarify the clinical relevance of these differences in cataract populations.

Discussion

Interpretation of the available literature is further complicated by differences in psychophysical testing methodologies. Farnsworth-Munsell and D-15 arrangement tests provide global patterns of chromatic confusion, whereas cone contrast testing and low-vision Cambridge Colour Test variants yield cone-specific thresholds that are particularly sensitive to S-cone-mediated tritan deficits. Similarly, Pelli-Robson and CSV-1000E charts offer single-score or limited sampling of the CS function, while quantitative CSF paradigms estimate the full contrast sensitivity function and are more sensitive to selective losses at mid-spatial frequencies. Variations in luminance, glare conditions, step size, and scoring algorithms across these instruments likely contribute to differences in reported effect sizes and complicate direct cross-study comparisons, underscoring the need for more standardized functional testing protocols in cataract research and clinical practice.

This review highlights that cataract-induced changes in the crystalline lens significantly impair both color vision and CS, which are critical for overall visual function and everyday activities [8,10,11,19,22]. The selective attenuation of short-wavelength light by lens yellowing explains the disproportionate effect on the tritan (blue-yellow) axis and the observed color desaturation, particularly in nuclear sclerotic cataracts [10,22,24,27]. These findings are consistent with psychophysical studies showing that S-cone-mediated contrast is preferentially reduced in the presence of lens brunescence and improves markedly after cataract extraction and intraocular lens implantation, supporting an optical rather than retinal or neural origin for much of the chromatic deficit [8,10,11,19,22].

The observation that CS deficits may be apparent even in the early stages of cataract formation, often preceding measurable acuity loss, underscores the inadequacy of relying solely on Snellen charts for clinical evaluation [8,9,11,19]. Quantitative contrast sensitivity function (qCSF) measurements reveal disproportionate losses at mid-spatial frequencies around 3-6 cycles per degree, a range strongly associated with real-world visual tasks such as object recognition, navigation in complex environments, and night driving [8,12]. Persistent CS loss in eyes with visual acuity of 20/25 or better suggests that these functional parameters are sensitive early indicators of cataract-related impairment and may better reflect patient-reported visual difficulties than high-contrast acuity alone [8,9,11,19].

From a clinical standpoint, these observations suggest that an acuity-centric approach alone may not fully capture the functional burden of cataract, and that CS, glare disability, and color vision testing may provide useful additional information in selected patients [8,9,11,12,19]. Patients who meet traditional visual acuity thresholds may nonetheless experience substantial limitations in driving, reading, and mobility if CS is significantly reduced or if blue-yellow discrimination is impaired [8,11,12,19]. Incorporating these functional metrics into clinical evaluation may help reduce the mismatch between measured visual acuity and patient-reported symptoms and may support more individualized decisions regarding surgical timing and intraocular lens selection [8,10,11,19,22,29,34].

The functional improvements observed after cataract surgery further reinforce the value of this multidimensional approach. Multiple studies have demonstrated rapid recovery of both color discrimination and CS following lens extraction, accompanied by significant gains in patient-reported quality of life [8,10,11,18,19,22,29,34]. Validated patient-reported outcome measures, such as the Catquest-9SF, further demonstrate that improvements in CS and color vision correlate strongly with enhanced quality of life following cataract surgery [17]. In many cases, postoperative enhancements in CS and glare tolerance are more closely aligned with patient satisfaction than improvements in Snellen acuity alone, highlighting the importance of including these endpoints when evaluating surgical success [8,9,11,12,19]. Nonetheless, the functional advantages of specific intraocular lens designs, such as blue-light-filtering, enhanced monofocal, and extended-depth-of-focus lenses, remain incompletely defined, with studies reporting small or inconsistent differences in CS and color vision between designs under various lighting conditions [16,37-41,43-46].

Current global eye health data indicate that cataracts remain a leading cause of avoidable visual impairment and blindness, particularly in low- and middle-income settings, and that the burden is expected to rise with population aging [1,4]. In this context, incorporating sensitive measures of functional vision into cataract care pathways may improve the identification of patients who would benefit most from timely surgery, even when acuity-based criteria are not yet met [1,4,8,12]. Adopting such an approach aligns with contemporary guideline recommendations that emphasize patient-centered outcomes, functional status, and quality of life alongside traditional clinical metrics [15]. In terms of health economics, cataract surgery is among the most cost-effective healthcare interventions globally, with substantial gains in functional independence and quality-adjusted life years [18]. These findings have multidisciplinary relevance for ophthalmology, optometry, geriatric care, and rehabilitation, as functional vision measures directly inform patient safety, clinical decision-making, and quality-of-life outcomes.

Limitations

The present review is limited by heterogeneity in cataract grading systems, psychophysical testing protocols, and outcome measures across the included studies. Differences in LOCS III implementation, contrast sensitivity charts, and color vision tests complicate direct comparisons and preclude quantitative pooling of data [8,9,11,19,21]. In addition, this work was conducted as a clinical review using a narrative synthesis approach rather than a formal systematic review framework or meta-analysis. Although the literature search was structured and focused on peer-reviewed English-language studies identified through PubMed and Scopus, the included literature may still be subject to publication and selection bias. Furthermore, most available evidence derives from cross-sectional or short-term postoperative studies, with relatively few longitudinal investigations tracking the long-term trajectory of functional recovery in color vision and contrast sensitivity after cataract surgery [8,10,18,19,29,34]. These limitations should be considered when interpreting the overall strength and generalizability of the reviewed evidence.

Future Directions

Future research should prioritize standardized, clinically feasible protocols for assessing color vision, contrast sensitivity, and glare disability in cataract patients. Standardized, validated testing protocols for color vision and contrast sensitivity, ideally integrated into digital platforms for consistency and ease of use, are urgently needed to facilitate cross-study comparisons and enable evidence-based guideline development [8,9,17]. Longitudinal studies with extended follow-up are needed to clarify the durability of postoperative gains and to identify factors associated with suboptimal functional recovery [8,10,18,19,29,34]. Additionally, comparative trials of emerging IOL designs-including blue-light-filtering, enhanced monofocal, and EDOF lenses-should incorporate robust functional endpoints, such as full contrast sensitivity functions and validated color vision tests, to clarify whether the reported differences between lens designs are clinically meaningful in routine cataract practice and to support more individualized lens selection [16,37-41,43-46]. Finally, advances in imaging and psychophysics, including adaptive optics and digital contrast sensitivity platforms, may provide deeper insight into the neural and optical substrates of cataract-related visual dysfunction and inform targeted interventions [8,9].

## Conclusions

Cataracts induce significant and differential impairment of visual function, depending on the type and severity of lens opacity. Nuclear cataracts predominantly affect color vision, whereas cortical and PSC cataracts are more closely associated with the loss of CS and glare disability. A comprehensive clinical evaluation that extends beyond visual acuity to include color discrimination and CS testing is essential for assessing functional impairments and guiding well-informed, patient-centered decisions regarding the timing of surgical intervention. Cataract surgery is a remarkably effective intervention that reliably restores these crucial visual functions, thereby significantly improving the quality of life of millions of patients worldwide.

## References

[1] (2026). World Health Organization: Blindness and vision impairment. https://www.who.int/news-room/fact-sheets/detail/blindness-and-visual-impairment.

[2] Bourne R, Steinmetz JD, Flaxman S (2021). Trends in prevalence of blindness and distance and near vision impairment over 30 years: an analysis for the Global Burden of Disease Study. Lancet Glob Health.

[3] Wan Z, Bai J, Wang W, Peng Q (2025). Global, regional, and national burden of cataract among older adults from 1990 to 2021: a comprehensive analysis based on the global burden of disease study 2021. Front Med (Lausanne).

[4] Cicinelli MV, Buchan JC, Nicholson M (2023). Cataracts. Lancet.

[5] Jiang B, Wu T, Liu W (2023). Changing trends in the global burden of Cataract over the past 30 years: Retrospective Data Analysis of the global burden of Disease Study 2019. JMIR Public Health Surveill.

[6] Vision Loss Expert Group of the Global Burden of Disease Study (2024). Global estimates on the number of people blind or visually impaired by cataract: a meta-analysis from 2000 to 2020. Eye (Lond).

[7] Flaxman SR, Bourne RRA, Resnikoff S (2017). Global causes of blindness and distance vision impairment 1990-2020: a systematic review and meta-analysis. Lancet Glob Health.

[8] Vingopoulos F, Kasetty M, Garg I (2022). Active learning to characterize the full contrast sensitivity function in cataracts. Clin Ophthalmol.

[9] Gupta L, Cvintal V, Delvadia R (2017). SPARCS and Pelli-Robson contrast sensitivity testing in normal controls and patients with cataract. Eye (Lond).

[10] Jolly JK, Pratt L, More AK (2022). The effect of cataract on color vision measurement with the low-vision Cambridge Colour Test: providing an adjustment factor for clinical trials. Ophthalmol Sci.

[11] Shandiz JH, Derakhshan A, Daneshyar A (2011). Effect of cataract type and severity on visual acuity and contrast sensitivity. J Ophthalmic Vis Res.

[12] Elliott DB, Gilchrist J, Whitaker D (1989). Contrast sensitivity and glare sensitivity changes with three types of cataract morphology: are these techniques necessary in a clinical evaluation of cataract?. Ophthalmic Physiol Opt.

[13] Owsley C, McGwin G (2010). Vision impairment and driving. Surv. Ophthalmol.

[14] Rubin GS, Adamsons IA, Stark WJ (1993). Comparison of acuity, contrast sensitivity, and disability glare before and after cataract surgery. Arch Ophthalmol.

[15] (2026). ESCRS: European Society of Cataract and Refractive Surgeons (ESCRS) recommendations for cataract surgery. https://www.escrs.org/escrs-guideline-for-cataract-surgery.

[16] Kara-Junior N, Espindola RF, Gomes BA (2011). Effects of blue light-filtering intraocular lenses on the macula, contrast sensitivity, and color vision after a long-term follow-up. J Cataract Refract Surg.

[17] Lundström M, Pesudovs K (2009). Catquest-9SF patient outcomes questionnaire: nine-item short-form Rasch-scaled revision of the Catquest questionnaire. J Cataract Refract Surg.

[18] Lamoureux EL, Fenwick E, Pesudovs K, Tan D (2011). The impact of cataract surgery on quality of life. Curr Opin Ophthalmol.

[19] Berríos-Dolz V, Chirinos-Saldaña P, Adrianzén RE (2020). Effect of cataract surgery on contrast sensitivity and quality of life in patients with different types of cataract. Rev. Mex de Oftalmol.

[20] Chylack LT Jr, Jakubicz G, Rosner B (1993). Contrast sensitivity and visual acuity in patients with early cataracts. J Cataract Refract Surg.

[21] Chylack LT Jr, Wolfe JK, Singer DM (1993). The lens opacities classification system III. Arch Ophthalmol.

[22] Mehta U, Diep A, Nguyen K (2020). Quantifying color vision changes associated with cataracts using cone contrast thresholds. Transl Vis Sci Technol.

[23] Friström B, Lundh BL (2000). Colour contrast sensitivity in cataract and pseudophakia. Acta Ophthalmol Scand.

[24] Langina-Jansone Z, Truksa R, Ozolinsh M (2020). Visual acuity and color discrimination in patients with cataracts. J Opt Soc Am A Opt Image Sci Vis.

[25] Iizuka T, Kawamorita T, Handa T, Ishikawa H (2023). Cone contrast test-HD: sensitivity and specificity in red-green dichromacy and the impact of age. J Opt Soc Am A Opt Image Sci Vis.

[26] Almustanyir A, Alhazmi M, Aldarwesh A (2025). Age-related effects on the color discrimination threshold. Life (Basel).

[27] Ao M, Li X, Qiu W (2019). The impact of age-related cataracts on colour perception, postoperative recovery and related spectra derived from test of hue perception. BMC Ophthalmol.

[28] Simunovic MP (2016). Acquired color vision deficiency. Surv Ophthalmol.

[29] Odinkemelu MC, Megwas AU, Azuamah YC (2023). Effect of cataract extraction with intraocular lens implant on contrast sensitivity, colour vision and photostress recovery time. Int J Res Rev.

[30] Singh B, Sharma S, Bharti N (2022). Visual and refractive outcomes of new intraocular lens implantation after cataract surgery. Sci Rep.

[31] de Souza RG, Golla A, Khan M (2022). Association of optical cataract indices with cataract severity and visual function. Int Ophthalmol.

[32] Abrahamsson M, Sjöstrand J (1986). Impairment of contrast sensitivity function (CSF) as a measure of disability glare. Invest. Ophthalmol. Vis. Sci.

[33] Bambo MP, Ferrandez B, Güerri N (2016). Evaluation of contrast sensitivity, chromatic vision, and reading ability in patients with primary open angle Glaucoma. J Ophthalmol.

[34] Zabel J, Michalak KP, Olszewski J, Przekoracka-Krawczyk A (2021). Color vision assessment following cataract surgery using anomaloscope. Ophthatherapy.

[35] McCann JJ, McCann MA (2019). Temporary tritanopia: Effects of cataract surgery on color. Color Imaging Conf.

[36] Pesudovs K, Burr JM, Harley C, Elliott DB (2007). The development, assessment, and selection of questionnaires. Optom Vis Sci.

[37] Hayashi K, Hayashi H (2006). Visual function in patients with yellow tinted intraocular lenses compared with vision in patients with non-tinted intraocular lenses. Br J Ophthalmol.

[38] Rodríguez-Galietero A, Montés-Micó R, Muñoz G, Albarrán-Diego C (2005). Blue-light filtering intraocular lens in patients with diabetes: contrast sensitivity and chromatic discrimination. J Cataract Refract Surg.

[39] Rodríguez-Galietero A, Montés-Micó R, Muñoz G, Albarrán-Diego C (2005). Comparison of contrast sensitivity and color discrimination after clear and yellow intraocular lens implantation. J Cataract Refract Surg.

[40] Leibovitch I, Lai T, Porter N (2006). Visual outcomes with the yellow intraocular lens. Acta Ophthalmol Scand.

[41] Cionni RJ, Tsai JH (2006). Color perception with AcrySof natural and AcrySof single-piece intraocular lenses under photopic and mesopic conditions. J Cataract Refract Surg.

[42] Queiroz MF, Ferreira FQ, Shimoda G (2023). Visual acuity, contrast sensitivity, and quality of life after bilateral implantation of multifocal diffractive intraocular lens. Arq Bras Oftalmol.

[43] Findl O, Leydolt C (2007). Meta-analysis of accommodating intraocular lenses. J Cataract Refract Surg.

[44] Kim DY, Park ES, Park H (2025). Comparative outcomes of the next-generation extended depth-of-focus intraocular lens and enhanced monofocal intraocular lens in cataract surgery. J Clin Med.

[45] Levy I, Shah RP, Mukhija R, Nanavaty MA (2025). Outcomes of mini-monovision with monofocal, enhanced monofocal and extended depth-of-focus intraocular lenses. Front Med (Lausanne).

[46] Mencucci R, Cennamo M, Venturi D (2020). Visual outcome, optical quality, and patient satisfaction with a new monofocal IOL, enhanced for intermediate vision: preliminary results. J Cataract Refract Surg.

[47] de Vries NE, Webers CA, Touwslager WR (2011). Dissatisfaction after implantation of multifocal intraocular lenses. J Cataract Refract Surg.

